# The effects of prostaglandins and endocannabinoids on iris arterial vascularization in Wistar rats - Experimental analysis

**Published:** 2019

**Authors:** Ioana-Cristina Coman, Mohammad Al Hammoud, Ruxandra Tudosescu, Raluca Iancu, Cosmina Barac, Cherecheanu Alina Popa

**Affiliations:** * Ophthalmology Department, University Emergency Hospital, Bucharest, Romania; ** Faculty of Medicine, ”Carol Davila” University of Medicine and Pharmacy, Bucharest, Romania; *** Medical Optics Ophthalmology Clinic, Dublin, Ireland; **** Ophthalmology Department, Regina Maria Private Clinic, Bucharest, Romania; ***** Ophthalmology Department, Braila Emergency Hospital, Romania

**Keywords:** NSAIDs, endocannabinoid system, glaucoma

## Abstract

**Introduction:** The iris vascular supply originates in the anterior and long posterior ciliary arteries. The endothelium influences local blood flow by releasing endothelium relaxing and contracting substances.

From a functional perspective, the ocular vascular tonus adjustment is humoral and neural dependent.

**Objectives:** The present article aims to evaluate the possible implications of topical administration of selective COX2 and nonselective COX inhibitors generically named nonsteroidal anti-inflammatory drugs (NSAIDs) and their possible interactions with the endocannabinoid system and the way they could interfere with the vascular tone at the level of ocular iris territory in Wistar rats.

**Materials and methods:** Experimental protocol on Wistar rats was performed in accordance with present laws regarding animal welfare and ethics in animal experiments (Directive 86/ 609EEC/ 1986; Romanian Law 205/ 2004; Romanian Laws 206/ 2004, 471/ 2002 and 9/ 2008; Romanian Order 143/ 400). The studied substances were instilled topically under general anesthesia, and images of the rat iris vessels were captured over a period of 10 minutes. The obtained images were further analyzed using an appropriate hardware and software program.

**Results:** The nonselective NSAIDs induced vascular dilation in the iris vessels, while the selective COX2 inhibitors determined a variable degree of vasoconstriction.

**Conclusion:** In view of the results of this experiment and the added evidence found in literature, we consider that further research will show the potential benefits for the additional use of NSAIDs in ocular pathology, otherwise unaffected by this medication until the present time (for example, glaucoma treatment).

## Introduction

The human iris and ciliary body are supplied by the anterior ciliary arteries along with posterior ciliary arteries and anastomoses from the anterior choroid [**1**].

The long ciliary arteries penetrate the globe without branching, explaining the higher pressure of the blood flowing through these arteries in comparison with the arterial pressure of the short ciliary arteries or retinal capillaries [**2**].

Besides the arteries subserving the ciliary processes and the iris, the major circle also provides some of the recurrent branches to the choroid. Thus, the two tissues comprising a portion of the anterior uvea share a common vascular supply [**2**,**3**].

From a functional perspective, the ocular vascular tone adjustment is humoral and neural dependent; for uveal and extraocular vessels, there is an important neural active adjustment, whereas the retina and optic nerve have control mechanisms involving only humoral adjustment [**4**,**5**]. The vasodilation is endothelium dependent [**4**].

Autonomic innervation of the ocular circulation is restricted to the vessels of the uvea and optic nerve; the retina appears to lack sympathetic and parasympathetic nerves [**6**,**7**]. Also, contrary to other vascular networks, the human retina lacks pre-capillary sphincters and therefore the retinal capillaries are continuously perfused [**7**].

The prostaglandins play an important role in the humoral adjustment mechanism. These autacoids generate a myriad of physiological and pharmacological actions in various organs of humans and animals [**8**,**9**]. The existence of TxA2 receptors in non-pigmented epithelial cells of the ciliary processes implies a possible role of TxA2 in some reactions of ocular inflammation, including the breakdown of blood aqueous barrier (BAB) and alteration of intraocular pressure [**10**,**11**].

Prostaglandin F2-alpha has a constricting effect on arteries; however, these effects can change according to drug concentrations, characteristics of the vascular bed, vascular size and animal species [**12**].

PGI-Synthase is constitutively expressed in endothelial cells, where it couples with COX-1. In vivo studies in mice and humans showed that COX-2 was the dominant source of PGI2 [**13**].

According to literature data, COX-2 is considered an inducible enzyme and does not seem to have a major effect on the vascular dynamic of the anterior pole [**14**,**15**]. 

The endocannabinoid system - derived from arachidonic acid via anandamide synthase – also elicits different vascular effects in certain vascular beds.

For the evaluation of the effects of different therapeutic substances with topical (ocular) or systemic administration, a carefully designed study of ocular vascularization is very important; as in present-day, a relatively wide array of topical antiglaucoma medication is available. In the past few years, many studies have been focused on the molecular mechanisms that control the disease process in glaucoma. This new approach strengthens our belief that glaucoma therapy beyond IOP lowering will become available.

## Aim 

This study aims to evaluate the possible implications of topical administration of selective COX2, nonselective COX inhibitors generically named nonsteroidal anti-inflammatory drugs (NSAIDs) and their possible interactions with the endocannabinoid system and the way they could interfere with the vascular tone at the level of ocular iris territory in Wistar rats. 

## Materials & Methods

For the experiments, male adult Wistar rats weighing 250 g to 350g were used, being brought into the laboratory with a minimum of three days before the experiments began and kept on a standard diet, with water and food supplied ad libitum. All the experiments were performed during daytime (9:00 AM to 6 PM) and conducted in a noise-attenuated environment. All animal procedures were carried out with the approval of the Local Ethics Committee of “Carol Davila” University of Medicine and Pharmacy, Bucharest, Romania, in accordance with the European Communities Council Directive 86/ 609/ EEC on the protection of animals used for scientific purposes, in accordance with present laws regarding animal welfare and ethics in animal experiments (Directive 86/ 609EEC/ 1986; Romanian Law 205/ 2004; Romanian Laws 206/ 2004, 471/ 2002 and 9/ 2008; Romanian Order 143/ 400).

Ibuprofen sodium salt, AM281 (CB1 cannabinoid receptor antagonist) and WIN55212-2 (CB1 and CB2 cannabinoid receptor agonist) were supplied by Sigma Aldrich. The other substances used were: Ketamine 5% (Calypsol 50mg/ ml produced by Gedeon Richter PLC HU), Pancuronium Bromide 2mg/ ml produced by Hospira UK LTD (GB), Diclofenac (Voltaren Ophtha CD 0.1% produced by Novartis Pharma Gmbh – DE), Meloxicam (Movalis vials 15mg/ 1.5ml produced by Boehrringer - ES), Parecoxib (Dynastat 20 mg produced by Pharmacia LTD- GB).

**Fig. 1:**
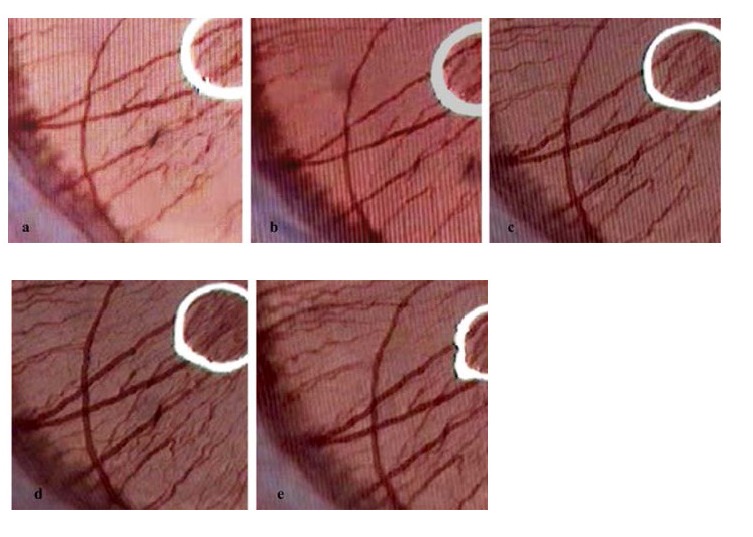
The aspect of LPCA diameter during topical administration of Ibuprofen 4.6%. Vascular diameter at: a - T0 (0 sec), b - T1 (30 sec), c - T2 (120 sec), d - T3 (310 sec), e - T4 (400 sec)

All rats were anaesthetized with Ketamine 5% - 100 mg/ kg body weight - injected intraperitoneally - while maintaining spontaneous respiration and the blink reflex; after five minutes Pancuronium Bromidum 0.02%, 0.1 mL/ 100 g body weight (injected intraperitoneally) was used to induce myorelaxation. Data recording was started after 10 minutes using a high magnification camera to image the vessels. After selecting the area of interest (long posterior ciliary artery – LPCA), manual adjustments of the image magnitude (maximum 400X), clarity and brightness were made and the experiment began. The image recording lasted 10 minutes; two instillations at 30 and 330 seconds were used. The test solutions were applied topically without touching the ocular surface. The temperature of substances instilled was 37°C. The first drug was saline or vehicle [mixture of Dimethyl sulfoxide (DMSO) and sodium chloride 0.5% - ratio 1:3], the second one was the active substance (Ibuprofen 1.15%, 2.3% ,4.6% Diclofenac 0.1%, Parecoxib 1%, 2%, 4%, Meloxicam 2.5%, 5%, 10%, WIN 55212-2 solution 0,2%, AM 281 solution 0,213% - equimolar doses only). Each subject served as its own control. The experiment design was parallel. The number of rats per group was 6, testing only the right eye (see **Fig. 1**).

The image acquisition system was composed of a CCD camera (Toshiba IK–642E) and an analog digital (AD) converter interface (Pinnacle micro Video DC10+) connected to an ASUS PC compatible system. The camera was fitted with a magnifying objective (Nikon) aided by an adapter (Navitar 1X Adapter 1–6015), allowing for resolutions within the optical microscopy range. Cold light was provided by a circular (ring-type fiber optics) source (Dolan– Jenner Industries Inc. model Fiber Lite series 180). The maximum optical resolution attained by the system was 12400 dpi (a pixel representing around 2×2 micrometers). The image analysis was carried out using VirtualDub and Adobe PhotoShop CS6, by measuring the variations of the vessels diameters before and after topic administration, at fixed time intervals: 0(T0i), 30(T1i), 120(T2i), 210(T3i), 300(T0), 330(T1), 420(T2), 510(T3), 600(T4) seconds (so, nine different measurements were made for each eye). The first value of vessels diameter, at 0 seconds (D0i) was considered the control value for the recording of each eye. Five diameters (mm) were measured at equidistant intervals of 10 pixels and the average value and standard deviation value were calculated for each data. Microsoft Excel was used for statistical processing of data. The comparison was made solely at the same target area according to initial conditions at 0s and 300s (e.g. T1i vs. T1, T2i vs. T2, etc). Thereby, we calculated the relative variations of the vascular diameter. 

Actual values (Da) were analyzed in relation to the initial value (D0i) by the following formulas: 

Vrel = (Da-Di0)/ Di0*100

For all the 6 rats, the values Vrel and the means standard error were analyzed using the T-test.

## Results

**Fig. 2:**
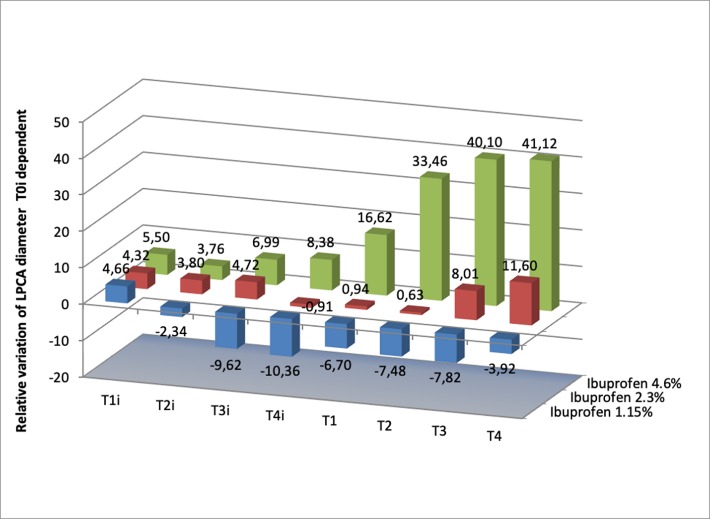
Relative variation (based on T0i value) of the vascular diameter of LPCA when using Ibuprofen 1.15%, 2.3% and 4.6%. Each value represents the percent of variation of vascular diameter at 30s (T1i), 120s (T2i), 210s (T3i), 300s (t4i), 330s (T1), 420s (T2), 510 s (T3), 600s (T4) standard error. The relative diameter at T1i for Ibuprofen 1.15% is 4.65±1.5, at T2i is -2.33±6.61, at T3i is -9.62±6.59, at T4i is -10.36±7.22, at T1 -6.70±8.40, at T2-7.47±8.46, at T3 is -7.82±8.24, at T4 -3.92±8.16; at 2.3% is 4.32±1.76, at T2i is 3.80±5.08, at T3i 4.72±4.57, at T4i -0.90±6.27, at T1 is 0.94±5.13, at T2 0.62±7.42, at T3 is 8±6.62, at T4 is 11.59±5.86 (p<0.05 vs. T0i value). The relative diameter at T1i for Ibuprofen 4.6% is 5.50±2.26, at T2i is 3.76±2.24, at T3i is 6.98±3.74, at T4i 8.38±3.17, at T1 is 16.61±6.43, at T2 33.46±11.54 (p<0.05 vs. T0i value), at T3 40.09±11.45(p<0.05 vs. T0i value), at T4 41.11±10.43 (p<0.05 vs. T0i value)

**Fig. 3:**
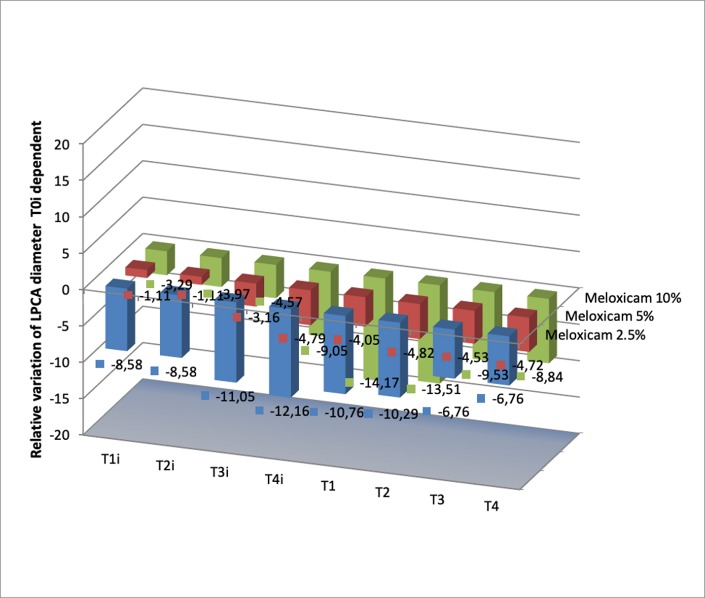
Relative variation (based on T0i value) of the vascular diameter of LPCA when using Meloxicam 2.5%, 5% and 10%; for Meloxicam 10% we obtained only one significant value (p<0.05 vs. T0i value) of the relative diameter at T1: -14.16±5.73

**Fig. 4:**
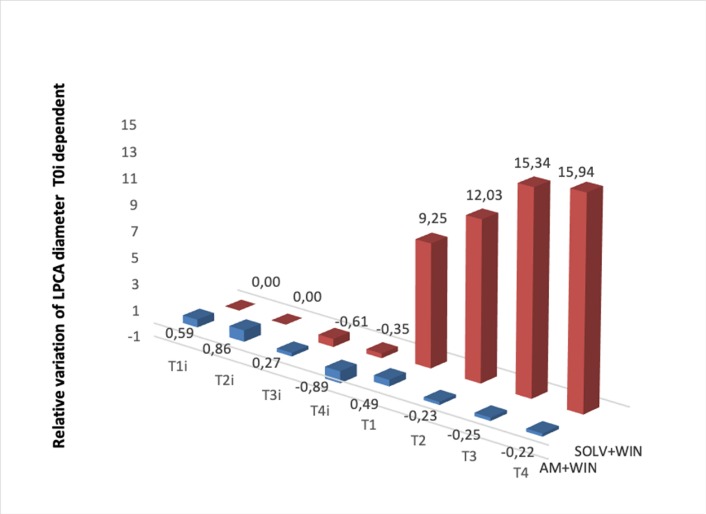
Comparative study of relative variation (based on T0i value) of the vascular diameter of LPCA when using **AM 281 0,213% + WIN 55212-2** 0,2% - (equimolar doses only) and vehicle (DMSO mixture of DMSO and sodium chloride 1:3 ratio) + WIN 55212-2 0,2%. It can easily be observed that AM 281 0,213% solution antagonized the vasodilator effect of WIN 55212-2 0,2% solution

**Fig. 5:**
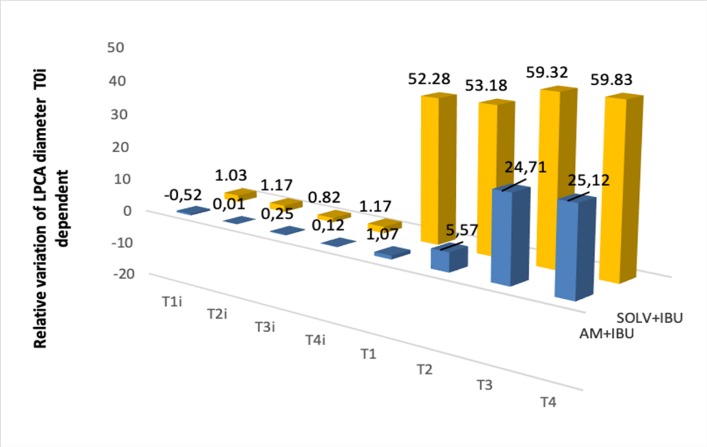
Comparative study of relative variation (based on T0i value) of the vascular diameter of LPCA when using **vehicle** (DMSO mixture of DMSO and sodium chloride 1:3 ratio) + **Ibuprofen 4.6% and AM 281 0,213% + Ibuprofen 4.6%**. Instillation of Ibuprofen 4.6% solution after DMSO mixture significantly increased arterial diameter with 52.82% at T1, 53.18% at T2, 59.32 % at T3 and 59.83% at T4; the vasodilation progressively increased from T1 (330 sec) to T4 (600 sec)

The results are presented in **Fig. 1**-**5**.

The saline solution used as negative control did not modify the vessel diameter. 

The three doses of Ibuprofen solution influenced the vascular reactivity differently. Thus, the lowest dose of Ibuprofen solution (1.15%) induced vasoconstriction from T1 to T4, with a maximum observed at T3. The higher doses (2.3% and 4.6%) induced vasodilation of LPCA in a dose-dependent manner, at all times measured.

Diclofenac 0.1% induced dilation of LPCA in a time-dependent manner. On the other hand, Meloxicam induced vasoconstriction at all doses used (see **Fig. 3**), whilst Parecoxib solution induced vasodilation only for the dose of 1%.

In terms of endocannabinoid system, WIN 55212-2 0.2% solution induced vasodilation beginning with the initial moment (T1), whereas AM281 0.212% solution (equimolar doses) antagonized the effect of WIN55212-2 (see **Fig. 4**). Afterwards, we studied the interactions between the Ibuprofen solution and the endocannabinoid system. The results are presented in **Fig. 5**. It can be observed that AM281 (selective CB1 receptor antagonist) partially antagonized the vasodilator effect of Ibuprofen 4.6%.

## Discussion

This study was undertaken because there is a relative lack of literature data evaluating the effect of topical administration of NSAID’s on anterior segment vascularization. 

Blood flow to the ciliary body includes both the ciliary muscle and the ciliary processes [**16**]. An advantage of this study is the use of Wistar rats with poorly pigmented iris allowing an easy visualization of the blood vessels. The substances investigated for their influence on iris blood flow were two NSAIDs, non-selective COX1/ COX2 inhibitors (Ibuprofen, Diclofenac), one relatively selective NSAID, COX2 inhibitor (Meloxicam) and one selective COX2 NSAID (Parecoxib). It is well known that cyclooxygenases are responsible for several types of prostaglandins which have either vasoconstrictor or vasodilator effect. If the iris vascular bed would present a prostaglandin tone, then decreasing the prostaglandin synthesis would induce changes of the vascular diameters. If the prostaglandin tone would be sustained by vasoconstrictor prostaglandins, their inhibition by Ibuprofen and Diclofenac should produce a vasodilator effect. If the prostaglandin tone were sustained by prostaglandins with vasodilator effect, then their inhibition by Ibuprofen should produce a vasoconstrictor effect. If this prostaglandin tone does not exist and the prostaglandins are involved solely in a phasic control, then topic administration of COX inhibitors should not change the vessels diameter. 

So, we are interested to shortly present the prostanoid system and its implications in ocular physiology and pathophysiology.

The humoral (paracrine) mechanism of vasoconstriction could be mediated via prostaglandin tone, supported by a higher concentration of thromboxane (TxA) receptors in the ciliary body. Binding sites for the thromboxane A2 (TxA2) receptor are primarily localized to the corneal epithelium, ciliary processes of the ciliary body, retina, and vessels surrounding the optic nerve. The binding sites for the TxA2 receptor in the anterior segment were mainly located on non-pigmented epithelial cells of the ciliary processes, whereas PGE2 and F2 alpha binding is mostly concentrated within the ciliary muscle; TxA2 causes vasoconstriction in practically all vascular beds [**10**].

The most important enzyme involved in prostaglandins and thromboxanes synthesis is COX, which exists as distinct isoform referred to as COX-1 and COX-2. The COX enzymes have 2 catalytic domains: peroxidase and cyclooxygenase active site [**17**,**18**].

High concentrations of PGF2-alpha have been reported to cause constriction in the ophthalmic and ciliary arteries of monkeys in vitro [**12**]. 

For our in vivo experiments, we chose the administration of two non-selective COX-1COX-2 inhibitors (Ibuprofen and Diclofenac) and two selective inhibitors of COX-2 (Meloxicam and Parecoxib), as well as AM281 - a potent and selective antagonist of CB1 cannabinoid receptor and WIN55212-2 - a potent cannabinoid receptor agonist.

For Ibuprofen, a dose/ effect relationship was demonstrated for the three tested doses; the highest vasodilator effect was obtained for 4.6%. We obtained the same results for Diclofenac, 0.1% - the single dose available on the market for topic administration. The results for Meloxicam showed vasoconstriction in all the three doses used (2.5%, 5% and 10%) (see **Fig. 3**), whilst Parecoxib solution induced vasodilation only for the dose of 1%. The other doses used produced a vasoconstrictor effect. 

This study demonstrates the presence of a prostaglandin-induced vasoconstrictor tone (at the level of LPCA), COX1-mediated and has been performed according to published literature, which shows that COX1 is the main functional enzyme in the platelets that couples preferentially with thromboxane synthase [**9**]. By contrast, COX-2 is the product of an immediate early gene that is rapidly inducible and tightly regulated [**19**,**20**].

According to literature, COX1 and COX2 do not have a uniform distribution in iris vascular bed of LPCA of Wistar rats; COX1 promotes thromboxanes synthesis and COX2 promotes prostacyclin synthesis [**14**]. We can conclude that COX1 inhibition promotes vasodilation in the iris vascular bed in groups treated with Diclofenac and Ibuprofen, while COX2 inhibition promotes a vasoconstriction to Meloxicam topic administration, which also applies in case of Parecoxib 2% in our study.

The most interesting results have been obtained for Ibuprofen. As it is well covered in literature, inhibition of prostaglandin synthesis is only one of the multiple effects of Ibuprofen, which also acts as a FAAH (Fatty Acid Amide Hydrolase) inhibitor. Through this mechanism, Ibuprofen might increase the local concentration of cannabinoids, their vasodilator effects achieving considerable notoriety by now (red eye to cannabis consumers).

In our study, topical administration of Ibuprofen determined a dose-dependent vasodilation. Also, according to our results, topical administration of WIN 55212-2 0.2% determined a significant vasodilation. The fact that WIN 55212-2 0.2% administered after AM 281 0,213% did not induce vasodilation allows the assumption that the CB1 cannabinoid receptors mechanism is involved in iris vascular tone regulation. AM281 single administration did not produce changes of vascular diameter, which led us to believe that the vascular regulation system is predominantly phasic, as opposed to the prostaglandin tonic regulation system. 

Ibuprofen solution (4.6%) increased vascular diameter by 52.28% at T1, 53.18 at T2, 59.32 at T3 and 59.83% at T4, while Ibuprofen 4.6% administrated after AM 281 0,213% increased the vascular diameter by 1.06% at T1, 5.57% at T2, 24.71% at T3 and 25.12% at T4. In both cases, the solution of Ibuprofen significantly increased the vascular diameter, showing that CB1 receptors inhibition did not antagonize the vasodilator effect of Ibuprofen completely. Thus, the vasodilator effect of Ibuprofen implies the local increase of cannabinoid receptors and also another mechanism like the decrease of vasoconstrictor prostaglandins tone.

## Conclusions

 Ibuprofen and Diclofenac increased the vessel diameter in the iris vascular bed.

Ibuprofen had a dose dependent vasodilator effect on LPCA.

The vasodilation induced by Ibuprofen is produced by two mechanisms: the decrease in vasoconstrictor prostaglandins tone and the increase of CB1 receptors availability.

Selective COX2 inhibitor administration did not increase the arterial diameter of LPCA in a dose dependent manner; in case of Meloxicam and Parecoxib (2.3%) we could observe a tendency towards vasoconstriction.

Considering COX1 as the main source of thromboxanes synthesis and also that its inhibition induces vasodilation, whereas prostacyclin synthesis is the main role of COX2, whose inhibition promotes vasoconstriction, we can state the hypothesis of a prostaglandin tone existence COX1-dependent [**9**,**21**].

There are at least 2 regulation mechanisms in the iris vascular bed: 

a) prostaglandin dependent regulation, which represents a tonic regulation system 

b) cannabinoid regulation, which represents a phasic regulation system.

The cannabinoid regulation from the iris vascular territory is CB1-mediated; the stimulation of CB1 receptors induces vasodilation.

## Final remarks

Some studies showed that TxA2 receptors are highly concentrated in the wall of the posterior ciliary arteries, suggesting that this receptor system could be involved in the pathophysiological mechanism of glaucomatous optic neuropathy [**10**]. Thus, we can conclude that this prostaglandin vasoconstrictor tone discovery could be an advantage for the future perspective treatments in glaucoma and its complications.

Another aspect is the secondary flow route of aqueous humor that comprises the vascular bed of the iris smooth muscles. Some NSAIDS could improve the non-trabecular outflow of aqueous humor by vasodilator effects [**22**]. 

Future studies will need to focus on the relationship between the vasoconstrictor prostaglandin tone and the administration of nonselective COX1/ COX2 NSAIDs, which could open new perspectives in glaucoma therapy.

**Conflict of interest**

The authors declare that they have no conflict of interest.
